# A single coiled‐coil domain mutation in hIKCa channel subunits disrupts preferential formation of heteromeric hSK1:hIKCa channels

**DOI:** 10.1111/ejn.16189

**Published:** 2023-11-29

**Authors:** James N. Charlick, Daniella Bozadzhieva, Andrew S. Butler, Kevin A. Wilkinson, Neil V. Marrion

**Affiliations:** ^1^ School of Physiology, Pharmacology and Neuroscience University of Bristol Bristol UK

**Keywords:** calcium‐activated, electrophysiology, heteromer, homomer, ion channel, subunits

## Abstract

The expression of IKCa (SK4) channel subunits overlaps with that of SK channel subunits, and it has been proposed that the two related subunits prefer to co‐assemble to form heteromeric hSK1:hIKCa channels. This implicates hSK1:hIKCa heteromers in physiological roles that might have been attributed to activation of SK channels. We have used a mutation approach to confirm formation of heterometric hSK1:hIKCa channels. Introduction of residues within hSK1 that were predicted to impart sensitivity to the hIKCa current blocker TRAM‐34 changed the pharmacology of functional heteromers. Heteromeric channels formed between wildtype hIKCa and mutant hSK1 subunits displayed a significantly higher sensitivity and maximum block to addition of TRAM‐34 than heteromers formed between wildtype subunits. Heteromer formation was disrupted by a single point mutation within one COOH‐terminal coiled‐coil domain of the hIKCa channel subunit. This mutation only disrupted the formation of hSK1:hIKCa heteromeric channels, without affecting the formation of homomeric hIKCa channels. Finally, the Ca^2+^ gating sensitivity of heteromeric hSK1:hIKCa channels was found to be significantly lower than the Ca^2+^ gating sensitivity of homomeric hIKCa channels. These data confirmed the preferred formation of heteromeric channels that results from COOH‐terminal interactions between subunits. The distinct sensitivity of the heteromer to activation by Ca^2+^ suggests that heteromeric channels fulfil a distinct function within those neurons that express both subunits.

## Graphical abstract

A single population of heteromeric channels with a distinct Ca^2+^ gating sensitivity was produced when wild‐type hSK1 and hIKCa subunits were co‐expressed in tsA201 cells. This was confirmed using a mutational approach. A single point mutation within the C‐terminal coiled‐coil domain of hIKCa subunits disrupted but did not prevent the formation of heteromeric channels, suggesting that heteromeric assembly is mediated by this region.

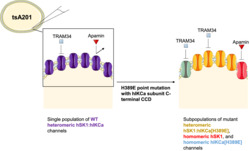

## 1 INTRODUCTION

Three types of small‐conductance Ca^2+^‐activated K^+^ channel (SK1–3) and one type of intermediate‐conductance Ca^2+^‐activated K^+^ channel (IKCa or SK4) have been cloned (Ishii et al., 1997; Joiner et al., 1997; Kohler et al., 1996). IKCa (SK4) channel subunits were originally proposed to be expressed in red blood cells and epithelial cells (Balut et al., 2012; Gardos, 1958). Cloning showed they possess approximately 40% identity with SK1–3 subunits and were originally termed SK4 (Joiner et al., 1997). However, they are now considered to be a separate subfamily (termed K_Ca_3.1; Balut et al., 2012; Brown et al., 2018; Turner et al., 2015) and are now more commonly termed IKCa (Higham et al., 2019; Ishii et al., 1997; Wulff et al., 2001). The belief that the physiological roles of SK1–3 and IKCa channels were distinct has been challenged in recent years by immunocytochemistry, electrophysiology and pharmacology showing IKCa subunit expression coincident with expression of SK1–3 channel subunits in some neurons (King et al., 2015; Sailer et al., 2002; Stocker & Pedarzani, 2000; Turner et al., 2015, 2016) and cardiac tissue (Tuteja et al., 2005; Weisbrod et al., 2013). This suggests a greater role for IKCa channel subunits in the body than has been previously considered. Any role might be more widespread when it is considered that it has been shown that human (h) SK1 subunits and IKCa subunits preferentially co‐assemble when co‐expressed in a heterologous expression system (Higham et al., 2019). The resulting heteromeric hSK1:hIKCa channel has a distinct single‐channel conductance and altered pharmacology. IKCa‐mediated current is blocked by the clotrimazole analog TRAM‐34 (Wulff et al., 2001). Block is mediated by association of TRAM‐34 with residues threonine 250 in the pore loop and valine 275 in transmembrane domain S6 (Wulff et al., 2001). Interaction with T250 might suggest that TRAM‐34 is an open‐channel blocker, although that has yet to be determined. It has been suggested that the pyrazole ring of TRAM‐34 uses the side chains of V275 on each subunit to be orientated for interaction with T250 of one subunit (Brown et al., 2018). This proposal suggests that high sensitivity to block of IKCa channel current by TRAM‐34 requires all four IKCa channel subunits. Co‐expression of SK1 and IKCa subunits produced a current that displayed a lower sensitivity to TRAM‐34 with a significantly reduced maximum block (Higham et al., 2019), supporting the mechanism of block of homomeric IKCa channel current.

SK channels assemble via interactions between coiled‐coil domains (CCDs) within the COOH terminus of each subtype (Church et al., 2015; Tuteja et al., 2010). CCDs are sequences that are implicated in protein–protein interactions, with the COOH termini of both SK and IKCa subunits displaying such domains close to the calmodulin‐binding domain (CaMBD) (Ji et al., 2018; Kim et al., 2007). Sequence analysis suggests that hIKCa subunits contain two separate COOH terminal CCDs, both displaying a histidine residue (H358 and H389). It has been suggested that histidine residues might be particularly important in protein function and assembly, with the phosphorylation state of H358 in hIKCa regulating channel activity (Srivastava et al., 2006).

We have used a mutation approach to investigate the preferred formation of heteromeric hSK1:hIKCa channels (Higham et al., 2019). Heteromeric channels containing hSK1 subunits mutated to be sensitive to the hIKCa inhibitor TRAM‐34 (Wulff et al., 2001) displayed a changed pharmacology when compared with co‐expression of wildtype subunits. These data, together with no evidence of homomeric channels, confirmed the preferential formation of heteromeric hSK1:hIKCa channels. Heteromeric channel formation was disrupted by a point mutation within the CCD in the COOH terminus of IKCa, the mutation not affecting homomeric channel formation. Finally, we demonstrated that the heteromer displays a calcium sensitivity distinct from homomeric hIKCa channels that could be exploited by the cell to optimize function.

## 2 METHODS

### 2.1 Cell culture and transient transfection

TsA201 cells were maintained in culture at 37°C and 5% CO_2_, being grown in culture flasks with Dulbecco's Modified Eagle Medium (DMEM) supplemented with foetal bovine serum (10%) and penicillin and streptomycin (1%). Cells were passaged at ~80% confluence. The cDNA of KCa channel subunits and one encoding expression of enhanced green fluorescent protein (eGFP) (using pcDNA3.1 or equivalent vectors) were transfected into tsA201 cells using polyethylenimine (PEI) (Merck/Sigma, UK) at a concentration of 0.2 mg/mL (Higham et al., 2019). Cells were used 48 h after transfection. All mutations were carried out using QuikChange II XL (Stratagene).

### 2.2 Electrophysiology

Outside‐out patches were excised from eGFP‐positive cells, pulled from thick‐walled (1.5 mm OD × 0.86 mm ID) borosilicate glass capillaries (Harvard Apparatus, UK), fire‐polished and filled with internal solution containing in mM: 97 K aspartate, 20 KCl, 1.5 Na_2_ATP, 10 EGTA, 9.65 CaCl_2_ (1 μM free Ca^2+^), 10 HEPES Na, 2.5 MgCl_2_ (pH 7.4 and osmolarity between 280 and 310 mOsm). Cells were bathed in, and patches excised into, an external solution containing in mM: 97 K Aspartate, 30 KCl, 10 HEPES Na^+^, 6.19 CaCl_2_ (1 μM free Ca^2+^), 1.44 MgCl_2_ (pH 7.4, 280–310 mOsm). Free Ca^2+^ concentrations were calculated using REACT (University of Strathclyde). Expressed channels were activated by the presence of 1 μM Ca^2+^ in the electrode solution and revealed by −100 mV to +100 mV voltage ramps (1 s duration) using Pulse v8.80 (HEKA). Current was measured using an Axopatch 200A amplifier, low‐pass filtered (cut off 1 kHz, Bessel, Frequency Devices) and acquired at 10 kHz (Pulse, HEKA). All recordings were made at room temperature. Apamin (Sigma‐Aldrich, UK), 6,12,19,20,25,26‐hexahydro‐5,27:13,18:21,24‐trietheno‐11,7‐metheno‐7*H*‐dibenzo [*b*,*n*] [1,5,12,16]tetraazacyclotricosine‐5,13‐diium dibromide (UCL1684; Tocris Biosciences, UK), and 1‐[(2‐chlorophenyl)diphenylmethyl]‐1*H*‐pyrazole (TRAM‐34; Tocris Biosciences, UK) were added to the external bath solution (superfusate) and applied at 10 mL^−1^ into a bath volume of approximately 1 mL. TRAM‐34 was dissolved in DMSO at 10 mM stock concentration, and aliquots were stored at −20°C until used. UCL1684 was dissolved at a stock concentration of 100 μM in DMSO, and aliquots were stored at −20°C. Finally, apamin was dissolved in distilled water at a stock concentration of 100 μM, and aliquots were stored at −20°C. All compounds were defrosted on the day of recording, with aliquots only thawed once, and desired concentrations were prepared by dilution in external solution.

Sensitivity of expressed channels to Ca^2+^ was assessed using inside‐out macropatches excised from eGFP‐positive cells, using electrodes of resistance 1–3 MΩ. A range of free Ca^2+^ concentrations was used, from 30 nM to 3 μM in half log_10_ increments. We used external solution to bath inside‐out patches, with the free Ca^2+^ concentration calculated to maintain free Mg^2+^ at 1 mM. Solutions were designed using REACT, with pH adjusted to 7.4 and osmolarity between 280 and 310 mOsm.

### 2.3 Data analysis

For concentration–inhibition relationships, data points representing inhibition of current amplitude were fit with a variable‐slope Hill equation in the following form:

$$\frac{I}{I_{\text{control}}}=A_{\min}+\left(\frac{A_{\max}-A_{\min}}{1+10^{\left({\text{LogIC}}_{50}-X\right)\times n_{H}}}\right)$$

where *I*_*control*_ is current amplitude in the absence of drug ((*X*), expressed in logarithmic units), *I* is current amplitude in the presence of drug, *A*_*min*_ is *I*_*min*_ divided by *I*_*control*_, *A*_*max*_ is *I*_*max*_ divided by *I*_*control*_, *IC*_*50*_ is the concentration of drug that blocks 50% of current that is sensitive to that drug, and *n*_*H*_ is the Hill coefficient (Graphpad Prism v9). Biphasic concentration–inhibition relationships were fitted using the following equation:

$$\frac{I}{I_{\text{control}}}=A_{\min}+\left(\frac{A_{\text{frac}}-A_{\min}}{1+10^{\left({\text{LogIC}}_{50,a}-X\right)\times n_{H,a}}}\right)$$

$$+\left(\frac{A_{\max}-A_{\min}}{1+10^{\left({\text{LogIC}}_{50,b}-X\right)\times n_{H,b}}}\right)$$

where *A*_*frac*_ is current amplitude at the maximum of the high‐sensitivity component *I*_*frac*_ */I*_*cont*_, *IC*_*50,a*_ is the IC_50_ of the high‐sensitivity component, *n*_*H,a*_ is the Hill coefficient of the high‐sensitivity component, *IC*_*50,b*_ is the IC_50_ of the low‐sensitivity component, and *n*_*H,b*_ is the Hill coefficient of the low‐sensitivity component. We provide different mean ± s.e.m. values for the IC_50_ obtained for inhibition of hIKCa‐ and hSK1:hIKCa‐mediated current by TRAM‐34 that were obtained by authors JNC and DB separately. There was no significant difference between values obtained by each experimenter (unpaired Student's *t* test). Statistical significance was determined using appropriate tests that are stated in the figure legend.

### 2.4 Structural modelling

The structure of homomeric hIKCa (SK4) in the calcium‐bound state (EMD‐7539) was used for all models (Lee & MacKinnon, 2018). The predicted structure of hSK1 subunits (Q92952; Jumper et al., 2021, Varadi et al., 2021) was used to generate homomeric hSK1 channels and heteromeric hSK1:hIKCa channels through alignment with the Cryo‐EM IKCa model. All models were created using PyMOL 2.6.0a0 (Schrödinger, LLC).

## 3 RESULTS

### 3.1 Confirmation of preferential formation of heteromeric hSK1:hIKCa channels

**FIGURE 1:**
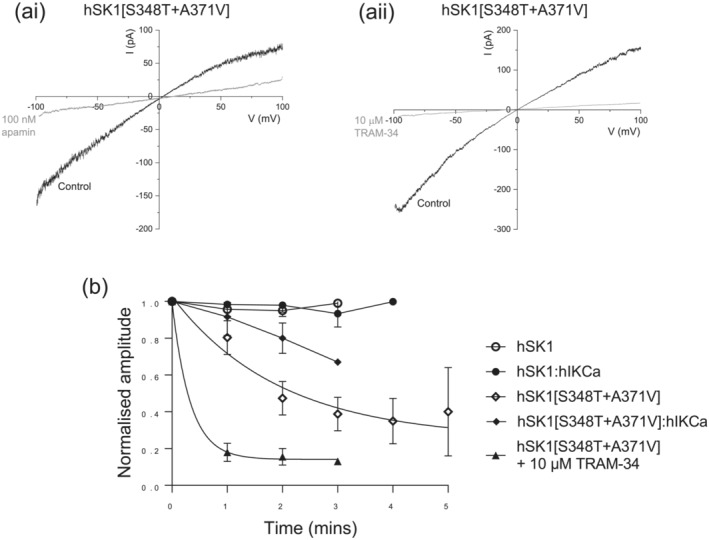
Generation of a pore mutant hSK1 subunit produced a current sensitive to both apamin and TRAM‐34. (ai) Current–voltage (IV) relationship for hSK1[S348T + A371V] generated by a voltage ramp from −100 to +100 mV (1 s duration) in the absence and presence of apamin (100 nM). The Ca^2+^‐dependent inward rectifying current was completely inhibited by apamin leaving a linear IV relationship. (aii) IV relationship for hSK1[S348T + A371V] in the absence and presence of TRAM‐34 (10 μM), showing the mutant hSK1 channel current was sensitive to the hIKCa inhibitor. (b) Diary plot showing amplitude of channel currents over time. Amplitude of hSK1‐ and hSK1:hIKCa‐mediated currents were stable, while current rundown was observed for both homomeric and heteromeric channels containing the hSK1[S348T + A371V] subunit. Homomeric hSK1[S348T + A371V]‐mediated current decreased in amplitude with a τ of 2 min (fit shown as solid line). Inhibition of hSK1[S348T + A371V]‐mediated current by TRAM‐34 (10 μM) was resolved by early application, causing inhibition of current with a τ of 20 s (fit shown as solid line).

We elected to utilise a change in pharmacology resulting from mutations in hSK1 to confirm the preferential formation of heteromeric channels when co‐expressed with hIKCa subunits. We introduced two point mutations within hSK1 (hSK1[S348T + A371V]) to attempt to make evoked current sensitive to block by TRAM‐34 (Wulff et al., 2001). Expression of hSK1[S348T + A371V] subunits produced functional current that displayed characteristic inward rectification and sensitivity to inhibition by extracellularly applied apamin (Figure 1ai). Our attempts to characterize the pharmacology of hSK1[S348T + A371V]‐mediated current was hampered by run‐down of evoked current (Figure 1b). Comparison of current amplitudes over time showed that in contrast to wildtype hSK1‐, wildtype hIKCa‐ or heteromeric wildtype hSK1:hIKCa‐mediated currents, both homomeric and heteromeric channel currents containing the hSK1[S348T + A371V] subunit displayed significant run‐down (Figure 1b). Construction of a diary plot illustrated that hSK1[S348T + A371V] decreased in amplitude, displaying a τ_rundown_ of 2 min (Figure 1b). Block of hSK1[S348T + A371V]‐mediated current was resolved by early addition of TRAM‐34, with current amplitude decreasing with a τ of approximately 20 s (Figure 1b). As predicted, introduction of the threonine (T250) and valine (V275) residues found in the hIKCa subunit sequence into hSK1 rendered the channel sensitive to block by TRAM‐34 (Wulff et al., 2001). In contrast to wildtype hSK1‐mediated current being insensitive to TRAM‐34 (Higham et al., 2019), hSK1[S348T + A371V]‐mediated current was fully blocked by 10 μM TRAM‐34 (Figure 1aii,b).

**FIGURE 2:**
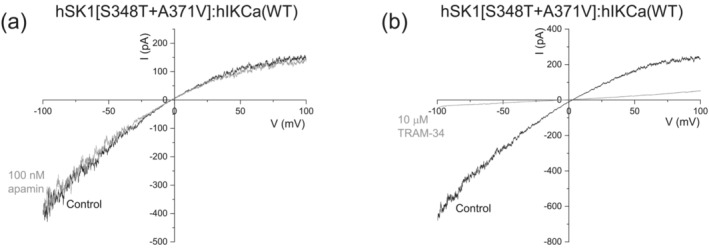
Expression of hSK1[S348T + A371V] with WT IKCa subunits produced a current that was insensitive to apamin but inhibited by TRAM‐34. (a) IV relationship generated by a voltage ramp from −100 to +100 mV (1 s duration) in the absence and presence of apamin (100 nM), showing that the heteromeric channel current is insensitive to the SK channel inhibitor. (b) IV relationship in the absence and presence of TRAM‐34 (10 μM), showing the current recorded from cells expressing both hSK1[S348T + A371V] and WT IKCa subunits was fully sensitive to the hIKCa inhibitor.

**FIGURE 3:**
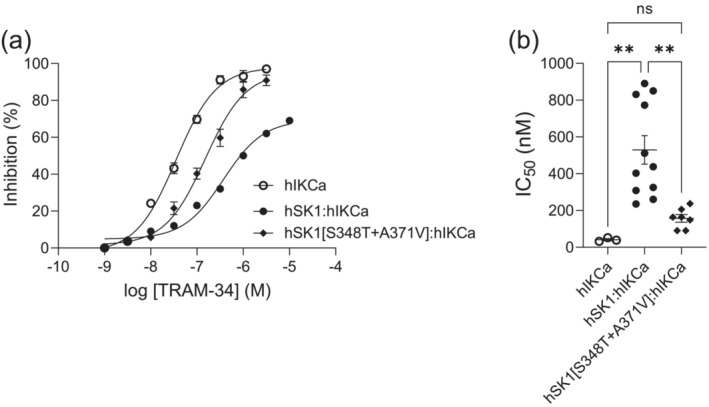
Comparison of sensitivities to inhibition by TRAM‐34. (a) Concentration–inhibition relationships generated by inhibition of expressed channel currents by increasing concentrations of TRAM‐34. Homomeric hIKCa‐mediated current was most sensitive and fully blocked by TRAM‐34. Current from cells expressing hSK1[S348T + A371V] and WT IKCa subunits was also fully sensitive to TRAM‐34, with an intermediate sensitivity between homomeric hIKCa‐ and heteromeric hSK1:hIKCa‐mediated current. (b) Mean IC_50_ values obtained from inhibition of each current subtype, with significance determined by one way ANOVA.

Co‐expression of hSK1[S348T + A371V] and hIKCa subunits produced current that displayed inward rectification and was insensitive to application of apamin (100 nM) (Figure 2a). The first observation that indicated inclusion of the hSK1[S348T + A371V] subunit within the expressed heteromeric channel was that current was fully sensitive to inhibition by TRAM‐34 (10 μM) (Figure 2b). This result is in contrast to sub‐maximal inhibition by TRAM‐34 (10 μM) of the wildtype hSK1:hIKCa heteromeric current (Higham et al., 2019) (Figure 3a). The sensitivity of homomeric and heteromeric current to inhibition by TRAM‐34 was compared by generation of concentration–inhibition relationships (Figure 3a). Homomeric hIKCa‐mediated current was fully inhibited by TRAM‐34, with an IC_50_ of 41 ± 5.9 nM (*n* = 3). As reported previously, TRAM‐34 produced only a sub‐maximal inhibition of heteromeric hSK1:hIKCa current, inhibiting current with an IC_50_ of 529.5 ± 77.3 nM (*n* = 11) (Higham et al., 2019). In contrast, TRAM‐34 maximally inhibited hSK1[S348T + A371V]:hIKCa heteromeric current with an IC_50_ of 157 ± 55 nM (*n* = 7). Figure 3b illustrates comparison of IC_50_ values showing a significant difference in values obtained for homomeric WT hIKCa, heteromeric WT hSK1:hIKCa and heteromeric hSK1[S348T + A371V]:hIKCa channel currents. These data confirmed that hSK1 and hIKCa prefer to form heteromeric channels.

### 3.2 A point mutation within the C‐terminal coiled‐coil domain of the IKCa subunit [H389E] permited homomeric channel assembly but disrupts preferential formation of heteromers with hSK1 subunits

It is proposed that assembly of both homomeric hSK and hIKCa channels is mediated by interactions between CCDs within the COOH terminal of each subtype (Church et al., 2015; Ji et al., 2018; Tuteja et al., 2010). hIKCa subunits contain two separate COOH terminal CCDs, with H358 in the first domain being able to regulate channel activity (Srivastava et al., 2006). The targeting of H389 would determine whether the second CCD domain is involved in assembly or function of homomeric or heteromeric channels.

**FIGURE 4:**
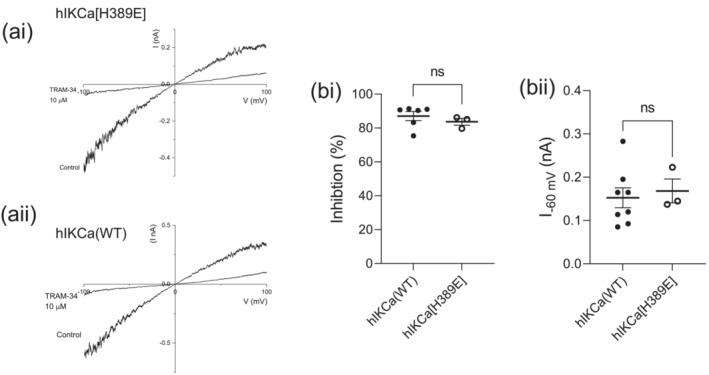
Expression of the hIKCa(H389E) subunit produced functional channels. (ai) IV relationship of expressed hIKCa[H389E]‐mediated current evoked by a voltage ramp from −100 to +100 mV (1 s duration) in the absence and presence of TRAM‐34 (10 μM). Evoked current was fully sensitive to TRAM‐34 (*n* = 3) and is shown in comparison with the effect of TRAM‐34 (10 μM) on WT IKCa‐mediated current (*n* = 6) (aii). (bi) Comparison of inhibition evoked by addition of 10 μM TRAM‐34 of both WT hIKCa‐ and hIKCa[H389E]‐mediated current. (bii) Comparison of excised outside‐out patch current amplitude measured at −60 mV for hIKCa(H389E) (*n* = 3) and WT hIKCa (*n* = 8) channels indicating comparable expression of both wildtype and mutant channels. Significance determined using unpaired Student's *t* test.

Expression of hIKCa[H389E] subunits gave rise to inwardly rectifying currents displayed in outside‐out patches excised from eGFP‐positive tsA‐201 cells (Figure 4ai). Current amplitude in excised patches was comparable with that observed with patches excised from cells expressing wildtype hIKCa (WT) subunits (Figure 4bii). Addition of TRAM‐34 (10 μM) produced 83.7 ± 2.0% (*n* = 3) block of hIKCa[H389E]‐mediated current (Figure 4ai), which was not significantly different (*p* = 0.437) from inhibition by TRAM‐34 (10 μM) of hIKCa (WT)‐mediated current (87.0 ± 2.6%) (*n* = 6) (Figure 4aii,bi). These data demonstrate that a reversal of net charge within the CCD of hIKCa did not affect assembly and function of homomeric hIKCa channels.

We used pharmacology to determine whether heteromeric channel formation was affected by the H389E mutation.

#### 3.2.1 Sensitivity to the hIKCa channel inhibitor TRAM‐34

**FIGURE 5:**
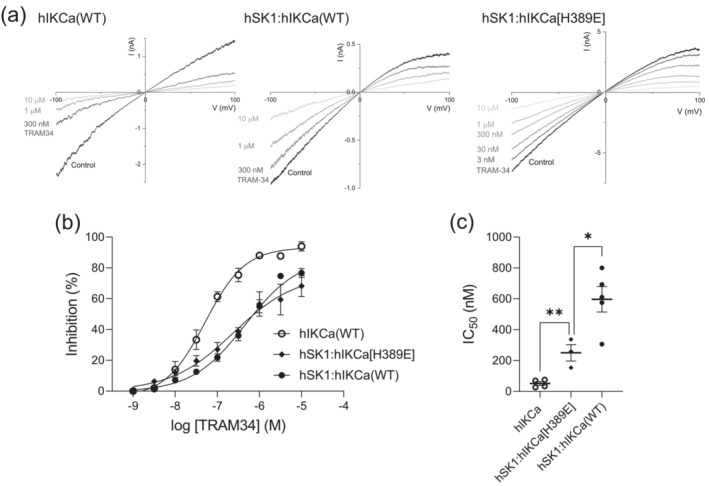
Sensitivities to inhibition by TRAM‐34 of homomeric and heteromeric channel currents. (a) IV relationships for hIKCa (left), hSK1:hIKCa (centre) and hSK1:hIKCa(H389E) (right) evoked currents in the absence and presence of increasing concentrations of TRAM‐34. (b) Concentration–inhibition relationships for the effect of TRAM‐34 on homomeric hIKCa, heteromeric hSK1:hIKCa and heteromeric hSK1:hIKCa[H389E] channel currents. Homomeric hIKCa‐mediated current was fully inhibited by TRAM‐34 with an IC_50_ of 51.1 ± 12 nM (*n* = 4), while both populations of heteromeric currents were only partially inhibited by TRAM‐34. The observed IC_50_ values for inhibition of heteromeric channel currents were 596.8 ± 82.4 nM for hSK1:IKCa (WT) (*n* = 4) and 250 nM ± 53 nM (*n* = 3) for hSK1:IKCa[H389E]. The higher sensitivity and less steep slope observed for inhibition of hSK1:hIKCa[H389E] channel current was suggestive of a mix of both homomeric and heteromeric channels. (c) IC_50_ values for homomeric hIKCa‐ and heteromeric hSK1:hIKCa‐mediated current and the comparison with the IC_50_ value from the inhibition relationship produced from cells co‐expressing hSK1 and hIKCa[H389E] subunits.

TRAM‐34 is a highly potent inhibitor of hIKCa channel‐mediated current (IC_50_ = 20–60 nM, Wulff et al., 2000, 2001) that does not affect hSK1‐mediated current (Higham et al., 2019). The high sensitivity of wildtype hIKCa channel‐mediated current to inhibition by TRAM‐34 was confirmed, displaying an IC_50_ of 51.1 ± 12.3 nM (*n* = 4) (Figure 5a,b). The effect of TRAM‐34 on heteromeric hSK1:hIKCa channel current has been previously shown to produce a sub‐maximal inhibition with a greatly reduced potency when compared with inhibition of wildtype hIKCa channel current (Higham et al., 2019) (Figure 5a,b). Application of increasing concentrations of TRAM‐34 incrementally inhibited current from cells co‐expressing hSK1 and hIKCa[H389E] subunits (Figure 5a). The concentration–inhibition relationship was best fit by a single Hill equation, with an increased sensitivity when compared with inhibition of heteromeric currents elicited by wildtype subunits (Figure 5b) (hSK1:IKCa [WT]: IC_50_ 596.8 ± 82.4 nM [*n* = 5]; hSK1:IKCa[H389E]: IC_50_ 250 ± 53 nM [n = 3]. *p* = 0.0247*). The leftward displacement and shallower slope of the concentration–inhibition relationship for cells co‐expressing hSK1 and hIKCa[H389E] subunits suggested that the curve represented the combined inhibition of both heteromeric hSK1:hIKCa[H389E] and homomeric IKCa[H389E] channels. We elected to use SK channel‐selective inhibitors to determine whether formation of hSK1:IKCa[H389E] heteromers was disrupted resulting in the presence of functional homomeric hSK1 channels.

#### 3.2.2 Sensitivity to the SK channel inhibitor apamin

**FIGURE 6:**
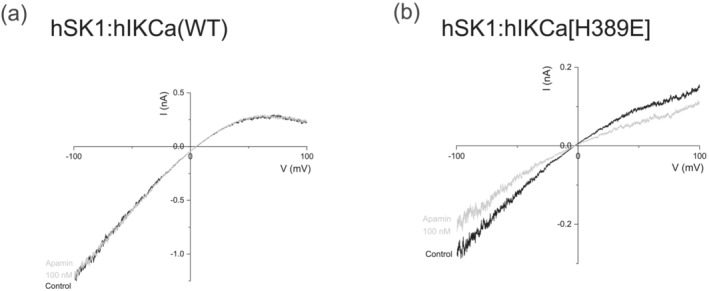
Disruption of heteromeric channel assembly reveals apamin‐sensitive hSK1‐mediated current. (a) Addition of apamin (100 nM) had no effect on current resolved from cells co‐expressing wildtype hSK1 and hIKCa subunits (*n* = 4). (b) Addition of apamin (100 nM) partially inhibited current resolved from cells co‐expressing wildtype hSK1 and the mutant hIKCa[H389E] subunits (*n* = 6).

Apamin does not affect current in cells co‐expressing hSK1 and hIKCa subunits, because of the preferential formation of heteromeric channels (Figure 6a) (Higham et al., 2019). In contrast, application of apamin (100 nM) to cells co‐expressing hSK1 and hIKCa[H389E] subunits reduced current amplitude by 17.3 ± 1.8% (*n* = 6) compared with the absence of inhibition of hSK1‐hIKCa (WT)‐mediated currents by apamin (*p* = 0.0003***) (Figure 6b). These data indicate the formation of homomeric hSK1 channels, suggesting that the point mutation within the COOH terminus of the hIKCa subunit disrupted preferential formation of heteromers.

#### 3.2.3 Sensitivity to the SK channel inhibitor UCL1684

**FIGURE 7:**
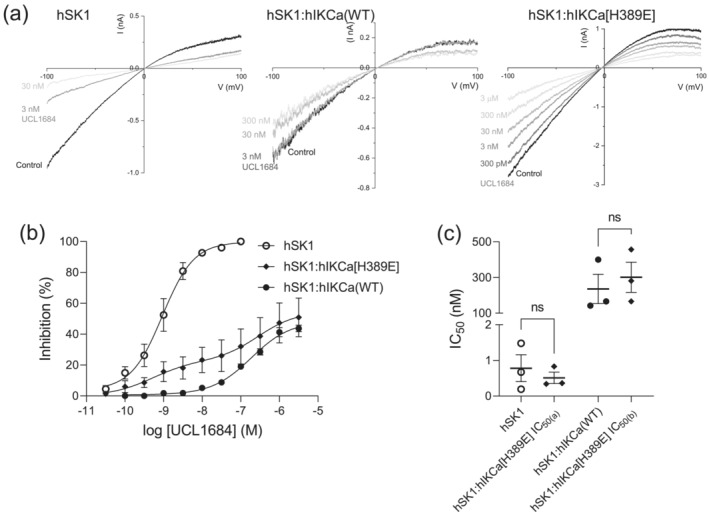
Homomeric hSK1‐mediated current identified by inhibition by UCL1684 resulting from disruption of heteromeric hSK1:hIKCa(H389E) channel formation. (a) IV relationships for hSK1 (left), hSK1:hIKCa (WT) (centre) and hSK1:hIKCa[H389E] (right) evoked currents in the absence and presence of increasing concentrations of UCL1684. (b) Concentration–inhibition relationships for the effect of UCL1684 on homomeric hSK1, heteromeric hSK1:hIKCa (WT) and heteromeric hSK1:hIKCa[H389E] channel currents. Homomeric hSK1‐mediated current was fully inhibited by UCL1684 with an IC_50_ of 783 pM (*n* = 3), while both populations of heteromeric currents were only partially inhibited by UCL1684. Heteromeric channels comprised of wildtype hSK1 and hIKCa (WT) subunits displayed a single component inhibition relationship with an IC_50_ of 236 nM (*n* = 3). In contrast, heteromeric channel current from cells co‐expressing wildtype hSK1 and mutant hIKCa[H389E] subunits displayed a two‐component inhibition relationship. The first component reflected inhibition of homomeric hSK1 channels (IC_50_ 511 pM), while the second component reflected inhibition of heteromeric hSK1:hIKCa[H389E] channel current (IC_50_ 301 nM) (*n* = 3). (c) Mean ± s.e.m IC_50_ values for homomeric hSK1‐ and heteromeric hSK1:hIKCa‐mediated current and the comparison with the IC_50_ values from each phase of the inhibition relationship produced from cells co‐expressing hSK1 and hIKCa[H389E] subunits. Significance was determined using the unpaired Student's *t* test.

Bis‐quinolinium cyclophane SK blockers with nanomolar potency have been designed, with UCL1684 being used commonly (Campos Rosa et al., 1998; Hancock et al., 2015). The original description of the preferential formation of hSK1:hIKCa channels reported that heteromeric current was relatively insensitive to UCL1684, with an approximately 10%–15% block of current observed with a high concentration (100 nM) of the SK channel blocker (Higham et al., 2019). We re‐evaluated this conclusion by determining whether expressed hSK1:hIKCa‐mediated current was sensitive to higher concentrations of UCL1684. Expressed heteromeric hSK1:hIKCa‐mediated current was clearly less sensitive to inhibition by UCL1684 than homomeric hSK1 current, with heteromeric current inhibited by high concentrations of UCL1684 (Figure 7a). The concentration–inhibition relationship illustrated that the current was sub‐maximally inhibited with an IC_50_ of 236 ± 82 nM (*n* = 3) (Figure 7b,c). In contrast, homomeric hSK1‐mediated current was maximally inhibited with an IC_50_ of 783 ± 380 pM (n = 3) (Figure 7b,c). We elected to use the <200‐fold difference in sensitivity between homomeric hSK1‐mediated and heteromeric hSK1:hIKCa‐mediated current to confirm that the mutation H389E disrupted heteromeric channel formation. Generation of the concentration–inhibition relationship for UCL1684 affecting expressed hSK1:hIKCa[H389E]‐mediated current showed a two component curve with maximal inhibition of 50.9 ± 12.5% that was significantly reduced when compared with inhibition of hSK1‐mediated current (*p* = 0.0171*) (Figure 7b). The IC_50(a)_ of 511 ± 0.2 pM and n_H(a)_ of 0.910 ± 0.02 (*n* = 3) of the high‐sensitivity component was not significantly different from the IC_50_ (*p* = 0.545) and n_H_ (*p* = 0.354) of UCL1684 inhibition of homomeric hSK1‐mediated current (Figure 7b,c). The IC_50(b)_ of 301 ± 84.6 nM and n_H(b)_ of 0.930 ± 0.1 (n = 3) of the low‐sensitivity component was not significantly different from the IC_50_ (*p* = 0.609) and n_H_ (*p* = 0.350) of UCL1684 on heteromeric hSK1:hIKCa‐mediated current (Figure 7b,c). These data confirmed that substitution of H389 by glutamate within the distal CCD of the hIKCa subunit disrupted either the formation or the stability of the heteromeric channel, resulting in a population of homomeric hSK1 channels being resolved.

### 3.3 Heteromeric hSK1:hIKCa channels were less sensitive to activation by Ca^2+^ than homomeric hSK1 and hIKCa channels

**FIGURE 8:**
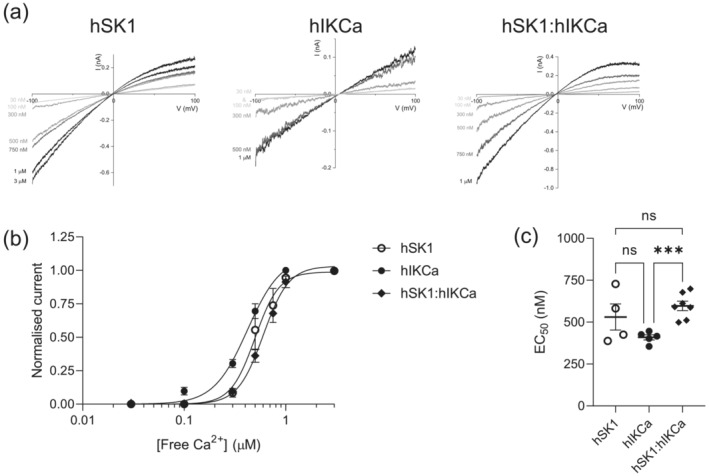
Differing sensitivities to activation by Ca^2+^ of homo‐ and heteromeric channels. (a) IV relationships for hSK1‐, hIKCa‐ and hSK1:hIKCa‐evoked currents in the presence of increasing concentrations of Ca^2+^. (b) Ca^2+^ activation curves showing that homomeric hIKCa channels appeared most sensitive to activation by Ca^2+^ (EC_50_ = 409 ± 15.3 nM) (*n* = 5), with a relationship that demonstrated that these channels were active at resting Ca^2+^ levels. In contrast, both homomeric hSK1 (EC_50_ = 531 ± 78 nM) (*n* = 4) and heteromeric hSK1:hIKCa (EC_50_ = 613 ± 27.6 nM) (*n* = 7) channels appeared less sensitive, with the Ca^2+^ dependence of heteromeric channels being consistent with those that underlie the hippocampal neuron slow afterhyperpolarization. (c) EC_50_ values for activation of hSK1, hIKCa and hSK1:hIKCa channels by Ca^2+^. There was no significant difference between EC_50_ values of hSK1 and hIKCa (*p* = 0.129) or hSK1 and hSK1:hIKCa channels (*p* = 0.359). However, there was a significant rightward shift of the EC_50_ value of hSK1:hIKCa channels compared with hIKCa channels (*p* = 0.0004***). Significance determined by one‐way ANOVA.

The activation of expressed homomeric hSK1 channels by Ca^2+^ has been shown to display EC_50_ values ranging from 300 to 700 nM (Hirschberg et al., 1998; Kohler et al., 1996; Xia et al., 1998). SK channels native to CA1 hippocampal neurons were activated with EC_50_ of 560 nM (Hirschberg et al., 1999). Activation by Ca^2+^ is highly cooperative, displaying Hill slopes between 3.5 and 5 (Hirschberg et al., 1998, 1999; Kohler et al., 1996; Xia et al., 1998). A range of sensitivity has been reported for activation of IKCa channels by Ca^2+^, with published EC_50_ values of 95 and 300 nM (Ishii et al., 1997; Joiner et al., 1997). Ramp currents evoked from inside‐out patches excised from tsA201 cells expressing hSK1 subunits exhibited Ca^2+^ dependence of activation. Homomeric hSK1‐mediated current was activated by Ca^2+^ with an EC_50_ of 531 ± 78 nM and a steep n_H_ of 5.26 ± 0.82 (*n* = 4). Activation of homomeric hIKCa‐mediated ramp currents was also Ca^2+^‐dependent, with an EC_50_ of 409 ± 15.3 nM and a less steep n_H_ of 4.10 ± 0.57 (*n* = 5). Finally, heteromeric hSK1:hIKCa‐mediated ramp currents showed Ca^2+^‐dependence, but with a right‐shifted EC_50_ of 613 ± 27.6 nM and a less steep n_H_ of 3.93 ± 0.29 (*n* = 7) (Figure 8a,b). There was a significant difference between the EC_50_ values obtained for activation of hSK1:hIKCa‐ and hIKCa‐mediated currents (*p* = 0.0004), but not between the values obtained for hSK1:hIKCa‐ and hSK1‐mediated currents (*p* = 0.359).

## 4 DISCUSSION

Mutating hSK1 to introduce the equivalent T250 and V275 residues found in hIKCa produced a homomeric hSK1‐mediated current that was now sensitive to the hIKCa inhibitor TRAM‐34. Co‐expression of the mutant hSK1 and wildtype IKCa subunits produced a heteromeric current of changed pharmacology when compared with co‐expression of wildtype subunits. Addition of apamin had no effect, clearly showing the lack of apamin‐sensitive homomeric hSK1 channels. These data confirmed that the co‐expression of hSK1 and hIKCa channel subunits produced the preferential formation of heteromeric channels.

**FIGURE 9:**
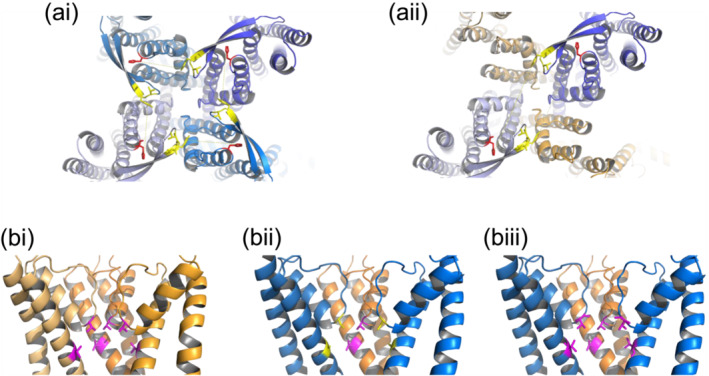
Apamin binding pocket is absent in heteromeric hSK1:hIKCa channels, while inclusion of the hSK1[S348T + A371V] subunit in heteromeric channels recreates the TRAM‐34 binding domain. (ai) Prediction of hSK1 channel structure by AlphaFold shows the donation of the S3–S4 extracellular loop (yellow) to the adjacent subunit, creating the two‐site binding motif for apamin (between loop [yellow] and outer pore [red]). (aii) Preferential formation of heteromeric channels produces a tetramer where subunits alternate in identity (hSK1, blue; hIKCa, orange). This arrangement of subunits places too great a distance between the S3–S4 extracellular loop and outer pore of the hSK1 subunits within the heteromeric tetramer, rendering the heteromeric channel insensitive to apamin. (bi) In wildtype hIKCa channels, T250 and V275 (magenta) form a TRAM‐34 binding pocket below the selectivity filter. (bii) Heteromeric channels preferentially formed upon co‐expression of wildtype hSK1 (blue) and hIKCa (orange) subunits disrupts this binding pocket and renders the heteromeric channel to be less sensitive to inhibition by TRAM‐34. (biii) Inclusion of the mutated hSK1[S348T + A371V] subunits within heteromeric channels recreates the TRAM‐34 binding pocket, increasing the sensitivity to inhibition by TRAM‐34.

Apamin is a negative allosteric inhibitor for SK1–3 channels that binds to the S3–S4 extracellular loop and outer pore to inhibit channel activity (Lamy et al., 2010), with the S3–S4 loop being donated to the adjacent subunit (Weatherall et al., 2011). The structure of hSK1 is predicted by using AlphaFold and shows the S3–S4 extracellular loop to be in a favourable position to be donated to the adjacent subunit (Figure 9ai) (Lee & MacKinnon, 2018). Current inhibition by apamin shows positive cooperativity, which has been shown to result from interactions between adjacent subunits (Lamy et al., 2010). Co‐expression of hIKCa and hSK1 subunits produces a heteromeric channel that is insensitive to apamin, which indicates that subunits must be alternately arranged within the tetramer (Higham et al., 2019). This arrangement places the S3–S4 extracellular loop and the outer pore of different hSK1 subunits too far apart to permit apamin binding. Construction of a model heteromer between hIKCa and hSK1 subunits illustrates how far apart the two binding sites for apamin will be (Figure 9aii). The arrangement of the four residues that are necessary for TRAM‐34 binding (Brown et al., 2018) is present in wildtype hIKCa channels (Figure 9bi) but absent in heteromeric hSK1:hIKCa channels (Figure 9bii). Recreation of this binding pocket through inclusion of hSK1[S348T + A371V] subunits in heteromeric channels (Figure 9biii) therefore produced a current inhibited by TRAM‐34 with the same sensitivity at wildtype hIKCa channels (Figure 3b).

We investigated whether the assembly of heteromers utilizes a CCD within the COOH terminus of the hIKCa channel subunit. We elected to investigate the role of H389 in subunit assembly, as protonation of the imidazole side chain of histidine residues at lower intracellular pH can be important in protein–protein interactions and assembly (Schonichen et al., 2013). Substitution of H389 within the hIKCa COOH terminal CCD with a glutamate (E) residue disrupted the preferred formation of heteromeric hSK1:hIKCa channels, without affecting the assembly of homomeric hIKCa channels. Finally, the functional consequences of heteromeric channel formation were investigated by measurement of the sensitivity to calcium of channel activation. We demonstrated that the heteromer displays a calcium sensitivity distinct from IKCa channels but similar to hSK1 channels. These data show that heteromeric channel formation utilizes a CCD within the COOH terminal of the hIKCa subunit that is different from that used to generate homomeric hIKCa channels. It is clear that the formation of heteromers produces a channel that displays a sensitivity to activation by calcium ions that will be exploited by the cell to optimize function.

It has been proposed that SK channels utilize the coiled‐coil domain (CCD) within the COOH terminus to assemble functional channels (Church et al., 2015; Ji et al., 2018; Tuteja et al., 2010). All SK channel subunits contain one CCD within their COOH termini, whereas the hIKCa subunit possesses two within its COOH terminus. The H389E mutation was introduced into the second (distal) CCD within the hIKCa C‐terminus. This mutation revealed the different structural requirements for assembly of homomeric and heteromeric IKCa channels, because it did not affect formation of homomeric hIKCa channels but did disrupt assembly of heteromeric channels. Resolution of an apamin‐ and UCL1684‐sensitive current in cells co‐expressing the mutant hIKCa and wildtype hSK1 subunits indicated the presence of homomeric hSK1 channels. It is interesting to note that the pharmacology of the largest current component in cells co‐expressing these subunits reflected the presence of heteromeric hSK1:hIKCa(H389E) channels. These data indicated that the H389E mutation did not prevent heteromer formation but only disrupted it. The H389E mutation would only disrupt the second C‐terminal CCD of hIKCa subunits and not the first. Functional homomeric hIKCa(H389E) channels were expressed, suggesting that assembly of homomeric channels is mediated by the first CCD, while heteromeric hSK1:hIKCa channel co‐assembly utilises the second COOH terminal CCD.

Co‐expression of hSK1 and hIKCa subunits produced preferential formation of heteromeric channels (Higham et al., 2019). Both subunits are expressed in the soma of hippocampal neurons (Bowden et al., 2001; Sailer et al., 2002; Turner et al., 2015) and the slow afterhyperpolarization (slow AHP) is somatic in origin (Lima & Marrion, 2007). The heteromeric channel shares pharmacological properties with the channel that underlies generation of the slow AHP (Higham et al., 2019; King et al., 2015). For example, hSK1:hIKCa channel current is sensitive to the analogs of clotrimazole (e.g., TRAM‐34) and charybdotoxin (Higham et al., 2019), as is the slow AHP (King et al., 2015; Shah & Haylett, 2000). In contrast, both hSK1:hIKCa‐mediated current and the slow AHP are insensitive to apamin (Church et al., 2019; Higham et al., 2019). These data indicate that homomeric SK1 channels do not underlie the slow AHP, whereas heteromeric hSK1:hIKCa channels are clearly a candidate channel to underlie generation of the slow afterpotential. The conductance of the channel underlying the slow AHP has been estimated to be in excess of 20 pS (Lima & Marrion, 2007), which is more consistent with the conductance of the heteromer rather than homomeric SK channels (Higham et al., 2019; Hirschberg et al., 1999). This conductance estimate for the channel underlying the slow AHP was derived from channels being activated by delayed facilitation of L‐type Ca^2+^ channels (Cloues et al., 1997; Sahu et al., 2017; Sahu & Turner, 2021). Non‐stationary noise analysis estimated an open probability (Po) of the channel at the peak of the slow AHP to be 0.6 (Valiante et al., 1997) and a very similar Po value was observed for the outward channels evoked by delayed facilitation of priming L‐type channels (Lima & Marrion, 2007). These properties led to the suggestion that a submembrane concentration of approximately of 0.5–1 μM Ca^2+^ would be required for this estimated Po (Marrion & Tavalin, 1998). The Ca^2+^ sensitivity of heteromeric hSK1:hIKCa channels (EC_50_ = 613 nM, inactive at 100 nM Ca^2+^) showed a tendency to be lower than the Ca^2+^ sensitivity of expressed homomeric hSK1 channels (EC_50_ = 531 nM, inactive at 100 nM Ca^2+^) and was significantly lower than homomeric hIKCa channels (EC_50_ = 409 nM, 10% active at 100 nM Ca^2+^). This lower sensitivity, together with the positive cooperativity of activation of hSK1:hIKCa channels by Ca^2+^, means that an increase of submembrane Ca^2+^ concentration would have an effect on heteromeric channel open probability. This allows for small changes in hSK1:hIKCa channel Po based on how much Ca^2+^ accumulates in the submembrane domain. In contrast, the higher cooperativity of homomeric hSK1 channel activation by Ca^2+^ would likely result in those channels being maximally activated. This is pertinent when it is considered that the amplitude of the slow AHP is dependent on the number of action potentials within the preceding burst (Church et al., 2019). This might reflect an increase in delayed facilitation of L‐type Ca^2+^ channels (Cloues et al., 1997), which will increase the likelihood of functional coupling between colocalised Ca^2+^ and outward channels (Marrion & Tavalin, 1998). This would also raise submembrane Ca^2+^ levels to increase outward channel open probability and increase the slow AHP amplitude. Therefore, it can be suggested that the pharmacology and Ca^2+^ dependence of activation of heteromeric hSK1:hIKCa channels places them as a strong candidate to underlie generation of the slow AHP.

The channel underlying the slow AHP is a therapeutic target for the treatment of cognitive impairment (Disterhoft et al., 1996; Moyer et al., 2000; Power et al., 2001), as the slow AHP amplitude increases with age (Disterhoft et al., 1996; Moyer et al., 2000; Power et al., 2001) and upon acute overexpression of those tau isoforms that are implicated in dementia (Stan et al., 2022). Resolution of the subunit identity comprising this elusive channel will be required to enable a new approach to target cognitive deficits in disease.

## AUTHOR CONTRIBUTIONS

**James N. Charlick:** Investigation; writing—review and editing. **Daniella Bozadzhieva:** Investigation. **Andrew S. Butler:** Methodology; writing—review and editing. **Kevin A. Wilkinson:** Methodology; project administration; writing—review and editing.

## CONFLICT OF INTEREST STATEMENT

The authors declare no conflicts of interest.

### PEER REVIEW

The peer review history for this article is available at https://www.webofscience.com/api/gateway/wos/peer-review/10.1111/ejn.16189.

## Data Availability

Data is available upon application.
